# Seismocardiography for nonexercise estimation of cardiorespiratory fitness in older adults

**DOI:** 10.1186/s12877-026-07771-6

**Published:** 2026-06-20

**Authors:** Magnus Sundberg, Anne Theil Gates, Carl-Johan Boraxbekk, Hartwig R Siebner, Jørn Wulff Helge, Casper Soendenbroe, Naiara Demnitz

**Affiliations:** 1 Danish Research Centre for Magnetic Resonance, Department of Radiology and Nuclear Medicine, Copenhagen University Hospital - Amager and Hvidovre, Copenhagen, Denmark; 2 Institute of Sports Medicine Copenhagen, Department of Orthopedic Surgery, Copenhagen University Hospital - Bispebjerg and Frederiksberg, Copenhagen, Denmark; 3 Department of Neurology, Copenhagen University Hospital Bispebjerg, Copenhagen, Denmark; 4 Institute for Clinical Medicine, Faculty of Medical and Health Sciences, University of Copenhagen, Copenhagen, Denmark; 5 Department of Biomedical Sciences, Faculty of Health and Medical Sciences, University of Copenhagen, Copenhagen, Denmark; 6 Department of Nutrition, Exercise and Sports, The August Krogh Section for Human and Molecular Physiology, University of Copenhagen, Copenhagen, Denmark

**Keywords:** V̇O₂-peak, Cardiorespiratory fitness, Older Adults, Seismocardiography, Ageing

## Abstract

**Introduction:**

Cardiorespiratory fitness predicts health and longevity. The gold-standard test for V̇O₂-peak, the cardiopulmonary exercise test (CPET), is difficult to conduct in older adults, and alternative methods offer low to moderate accuracy. This project compared a new method, seismocardiography, to CPET in older adults. It was hypothesized that seismographic V̇O₂-peak estimates would strongly correlate with CPET-derived V̇O₂-peak. Method: Thirty-six healthy older adults (aged 65–75 years; 19 female) completed the seismocardiography assessment (Seismofit^®^) of resting V̇O₂-peak and a CPET on a cycle ergometer. Two criteria were used to support the attainment of maximal oxygen uptake (V̇O₂-peak) during the ramp exercise test: (1) a V̇O₂ plateau, defined as an increase in oxygen uptake of less than 150 mL·min⁻¹ despite a further increase in work rate during the final phase of the incremental protocol, and (2) a respiratory exchange ratio (RER) > 1.15. The V̇O₂-peak estimation at rest was performed over 40 s with participants lying supine while wearing a Seismofit^®^ chest sensor (VentriJect, Hellerup, Denmark). Concordance between measurements was assessed using correlations and Bland-Altman plots, and paired t-test evaluated mean differences between Seismofit and CPET-derived V̇O₂-peak.

**Results:**

The sample had a mean age of 70 ± 3.0 years and BMI of 25.7 ± 2.8 kg/m². For all participants, a significant positive correlation was observed between Seismofit^®^ and measured V̇O₂-peak (*r* = 0.57, *p* < 0.001). When restricting the analysis to the 27 participants with validated V̇O₂-peak, a strong significant positive association between the methods was found (*r* = 0.80, *p* < 0.001). For all participants, Seismofit significantly overestimated V̇O₂-peak (t = 4.48, df = 35, *p* < 0.001).

**Conclusion:**

This study demonstrated that Seismofit show a moderate-to-strong association with CPET measurements in older adults. Seismofit, however, systematically overestimated V̇O₂-peak. For Seismofit to be integrated into large-scale cohort studies and experimental research, the methodology used for older adults needs further development.

**Supplementary Information:**

The online version contains supplementary material available at https://doi.org/10.1186/s12877-026-07771-6.

## Introduction

With rising life expectancy, a growing number of older adults experience age-related impairments such as cognitive decline [1], hypertension [2], cardiovascular disease [3], and functional impairments [4]. Cardiorespiratory fitness (CRF) is a strong predictor of health and longevity, closely linked to reduced risk of chronic diseases, functional decline, and cognitive ageing [5, 6].

An important indicator of CRF is the gold standard measurement of maximal oxygen uptake (V̇O₂-peak) during a graded cardiopulmonary exercise test (CPET), commonly performed on a cycle ergometer or treadmill [7]. From a public health perspective, accurate assessment of V̇O₂-peak is crucial for tailoring strategies that promote healthy ageing at the individual level. However, accurate determination of CRF by reaching V̇O₂-peak requires exercising to volitional exhaustion, which can be particularly challenging for older adults due to age-related impairments that limit their ability to perform incremental exercise. Although CPET can be feasible in this population, it may raise concerns about burden, safety, and tolerability [8]. Additionally, CPET requires specialized equipment and trained personnel to administer and interpret the test, which further limits its feasibility in many settings [7]. Accordingly, despite its value, direct V̇O₂-peak measures in large ageing studies and/or clinical settings are often limited. For example, large cohorts such as the UK Biobank and the Health and Retirement Study have conducted submaximal exercise protocols or step-tests instead of CPET due to feasibility and cost [9, 10]. While these methods are easier to administer, they generally offer only low to moderate accuracy compared to CPET [11]. Overall, a simpler and more affordable method for estimating V̇O₂-peak would facilitate its inclusion in large-scale cohort studies and experimental research, providing a more complete picture of how cardiorespiratory fitness influences ageing and public health.

Notably, a non-invasive approach called Seismofit^®^, which uses seismocardiography (SCG) to estimate V̇O₂-peak, has recently emerged. Seismofit provides a V̇O₂-peak estimate in 40 s while lying still, without physical demands. The Seismofit approach estimates V̇O₂-peak via seismocardiography and was developed and tested against CPET in healthy adults, showing strong correlations (*r* = 0.80 and *r* = 0.90 after adjustments for age, sex, and BMI) [12]. A recent study comparing Seismofit against CPET in young adults and athletes, showing a strong correlation with the gold-standard measures (*r* = 0.875) [13]. Crucially, validation of SCG in older adults is lacking. The present study aims to determine whether Seismofit provides an accurate V̇O₂-peak estimate in older adults. We hypothesized that SCG-derived V̇O₂-peak estimates would also be strongly correlated with gold-standard V̇O₂-peak values in healthy older adults. In addition, subgroup differences, including sex, age, and fitness level, were explored to assess whether SCG-derived V̇O₂-peak estimates perform efficiently across these groups.

## Method

### Study design

This study used baseline data from the HIT-ing the neurometabolic targets for exercise-induced effects on cognition (HINT) randomised controlled trial study, which tested the acute effects of exercise on cognitive function and brain metabolites using magnetic resonance imaging (MRI) in older adults (study protocol: https://osf.io/nueth/). In the HINT trial, participants completed three study visits between 2023 and 2025. The first visit included assessments of participant characteristics, cognitive performance, and fitness level, all conducted at the Institute of Sports Medicine Copenhagen, Bispebjerg Hospital. To evaluate the participants’ fitness levels, they completed an SCG assessment followed by a CPET. For the present study, only data from the first study visit were included. Ethical approval was obtained from the Danish Scientific Ethics Medical Committees (2301171). The study was conducted in line with the Declaration of Helsinki.

### Study sample

Only participants who completed both the SCG assessments and the CPET assessment were included in the present analysis. Of the 48 healthy older adults included in HINT, the first 12 participants did not complete SCG assessments. Participants were recruited from the Greater Copenhagen area through community centers, online advertisement (e.g., ForskningNu) and social media platforms. To be eligible for participation, participants had to be free from: cardiac or pulmonary diseases, musculoskeletal or orthopedic limitations, neurological illnesses or any contraindications for MRI scanning, such as metallic implants or claustrophobia. Participants had to be between 65 and 75 years of age and engage in no more than two bouts of vigorous physical activity per week. Vigorous physical activity was defined as activities requiring high effort and a significant increase in heart rate and respiration. Screening was conducted by a sports scientist who would consider each individual activity reported by the participant. All participants received both written and verbal information about the study before providing written informed consent. Sex, age, height, and weight were recorded. BMI was calculated using height and weight. Physical activity level was determined using the self-report questionnaire, Physical Activity Scale for the Elderly (PASE) [14].

Based on CPET-measured V̇O₂-peak, participants were categorized into different fitness levels based on data from Eriksen et al., (2016) [15]. Using the mean V̇O₂-peak levels for each sex and age group, females aged 65–69 years were categorized into low (< 26.5 ml/min/kg) and high fitness levels (≥ 26.5 ml/min/kg). Females aged 70 years or above were categorized into low (< 24.1 ml/min/kg) and high fitness levels (≥ 24.1 ml/min/kg). Males aged 65–69 years were categorized into low (< 33.2 ml/min/kg) and high fitness levels (≥ 33.2 ml/min/kg). Males aged 70 years or above were categorized into low (< 29.6 ml/min/kg) and high fitness levels (≥ 29.6 ml/min/kg). For the exploration of sub-groups, participants were divided into low and high fitness groups. Furthermore, the participants were also split by sex and age (younger < 70 years and older 70 ≥ years).

### Measures

#### SCG-Derived V̇O₂-peak

Seismofit (VentriJect, Copenhagen) was used to estimate participants’ V̇O₂-peak. The Seismofit consisted of a chest-mounted medical device with a 3-axis digital accelerometer, a smartphone app, and a cloud-based signal processing solution. Seismofit uses seismocardiography to estimate V̇O₂-peak at rest [16]. The Seismofit sensor was first placed approximately 2 cm from the sternum and secured with double-sided adhesive skin tape. Sex, age, height, and weight were recorded and entered into an application connected to the monitor (App: VentriJect). Participants were then asked to lie down and rest on a hospital bed for five minutes. During this time, they were instructed to relax. After five minutes, the recording was started through the application. The monitor recorded for 40 s and estimated V̇O₂-peak based on these signals, along with the participant’s age, sex, height, weight, and heart rate during the 40-second recording [16]. The heart rate is measured with an accelerometer inside the Seismofit device. Only a single recording was conducted. The equation model Seismofit uses to estimate VO₂-peak has been described previously [16].

#### Cardiorespiratory fitness testing

After the seismocardiography protocol, the participants completed a CPET to determine their V̇O₂-peak. The tests were performed on an ergometer bike (Corival/CPET, Lode). A standard 12-lead electrocardiogram (ECG) was recorded throughout the test to ensure safety and to monitor the participant’s heart rate. The ECG electrodes were placed to accommodate movement during exercise. Before each CPET session, the metabolic cart was calibrated following the manufacturer’s guidelines, including calibration procedures for the gas analyzer and automatic flow/volume. CPET was performed using an individualized continuous-ramp protocol on a cycle ergometer. The protocol was individualized for each participant based on their weekly physical activity, sex, and weight to predict their target end power output (in watts). Ramp slopes were selected by the sports scientist conducting the tests based on each participant’s predicted maximal work rate, with the aim of reaching this workload about 10 min into the incremental phase. Participants expected to reach higher peak workloads received steeper ramp slopes. Workload increased continuously each second throughout the test. All participants began with a 3-minute warm-up at 5 watts, followed by the incremental phase. Participants were required to maintain a cadence between 60 and 100 revolutions per minute (RPM), and the test was designed to reach V̇O₂-peak within 8 to 12 min [17]. Participants have been instructed to maintain a steady pace during the test to ensure the workload remains consistent. Oxygen consumption was measured using a breath-by-breath gas analysis system (Vyntus CPX, Intra Medic). Two criteria were used to support the attainment of maximal oxygen uptake (V̇O₂-peak) during the ramp exercise test: (1) a V̇O₂ plateau, defined as an increase in oxygen uptake of less than 150 mL·min⁻¹ despite a further increase in work rate during the final phase of the incremental protocol, and (2) a respiratory exchange ratio (RER) > 1.15 [18]. To ensure an accurate comparison with SCG estimates, a validated CPET sample was defined as measurements that met all criteria for attaining V̇O₂-peak.

### Statistical analyses

All statistical analyses were conducted in RStudio (version 2023.12.1–402), running on R version 4.3.2 (2023-10-31). The mean and SD of all outcome variables were calculated. Before conducting any statistical analyses, the normality of the data was assessed using the Shapiro–Wilk test, and distributions were visually inspected using histograms and Q–Q plots. Homoscedasticity and linearity were evaluated by examining scatterplots of residuals versus fitted values. Pearson correlation analyses were performed to examine associations between estimated V̇O₂-peak from Seismofit (henceforth Seismofit) and measured V̇O₂-peak from the CPET (henceforth V̇O₂-peak). Correlation coefficients were interpreted using the following thresholds: *r* < 0.40 was considered weak, 0.40–0.69 moderate, and ≥ 0.70 strong [19]. The Bland–Altman method was used to compare Seismofit estimates with CPET measures and to evaluate the level of agreement between the two methods. Additionally, comparison between Seismofit and CPET-derived V̇O₂-peak was assessed using paired t-tests, Cohen’s d effect size, mean absolute percentage error (MAPE), and the standard error of the estimate (SEE). Analyses were conducted on the whole dataset and then repeated on those individuals who satisfied all CPET criteria (i.e. Validated CPET Sample). Significance was defined as *p* < 0.05.

Post-hoc analyses explored the role of age group (< 70 years vs. ≥ 70 years), sex, and fitness level (low vs. high) in the relationship between CPET and Seismofit estimates, using linear models with interaction terms and stratified sub-group analyses. The subgroup analyses were conducted for exploratory purposes.

## Results

In this study, 36 participants were included (Table 1). The mean age was 70 ± 3.0 years, with a mean BMI of 25.7 ± 2.8 kg/m². The participants had a mean maximum heart rate during CPET of 155 ± 10 beats per minute (BPM), a CPET measured V̇O₂-peak of 26.5 ± 4.9 ml/min/kg, and an SCG-estimated V̇O₂-peak of 30.3 ± 6.0 ml/min/kg. SCG-estimated V̇O₂-peak was on average 14.6% higher than CPET-measured V̇O₂-peak.

**Table 1:** Participants characteristics and fitness measurements

	Full Sample (*n* = 36)	Validated CPET Sample (*n* = 27)
Participant Characteristics		
Sex (*n* female, %)	16, 44	16, 59
Age (years)	69.7 ± 3.1	70.3 ± 3.2
Height (cm)	168.3 ± 8.8	168.8 ± 8.8
Weight (kg)	73.1 ± 14.9	74.3 ± 15.4
BMI (kg/m2)	25.7 ± 3.8	26.1 ± 4.2
Fitness Measurements		
Maximum Heart Rate (BPM)	154.9 ± 10.5	156.1 ± 9.8
V̇O₂-peak (ml/min/kg)	26.5 ± 4.9	26.9 ± 5.1
RER at Exhaustion	1.3 ± 0.8	1.3 ± 0.1
Seismofit (ml/min/kg)	30.3 ± 6.0	28.8 ± 5.9
PASE	128.6 ± 53.6	130.5 ± 53.9

A paired-samples t-test was conducted to assess the differences between Seismofit and cardiopulmonary exercise testing (CPET)-derived V̇O₂-peak. For all participants, Seismofit significantly overestimated V̇O₂-peak (t = 4.48, df = 35, *p* < 0.001), with a mean difference of 3.87 ml/kg/min and a 95% confidence interval of 2.11 to 5.62 ml/kg/min. The effect size was moderate (Cohen’s d = 0.69, 95% CI: 0.35 to 1.04), indicating a meaningful systematic difference between methods. Prediction error was moderate, with an MAPE of 20.09% and a SEE of 5.05 ml/kg/min.

For participants who reached a validated V̇O₂-peak, Seismofit still significantly overestimated V̇O₂-peak (t = 2.71, df = 26, *p* = 0.012), although the magnitude of the difference was reduced to 1.85 ml/kg/min (95% CI: 0.45 to 3.25 ml/kg/min) with a small effect size (Cohen’s d = 0.33, 95% CI: 0.08 to 0.58). Prediction accuracy improved, with MAPE decreasing to 12.66% and SEE to 3.59 ml/kg/min.

### Correlation analyses

**Fig. 1:**
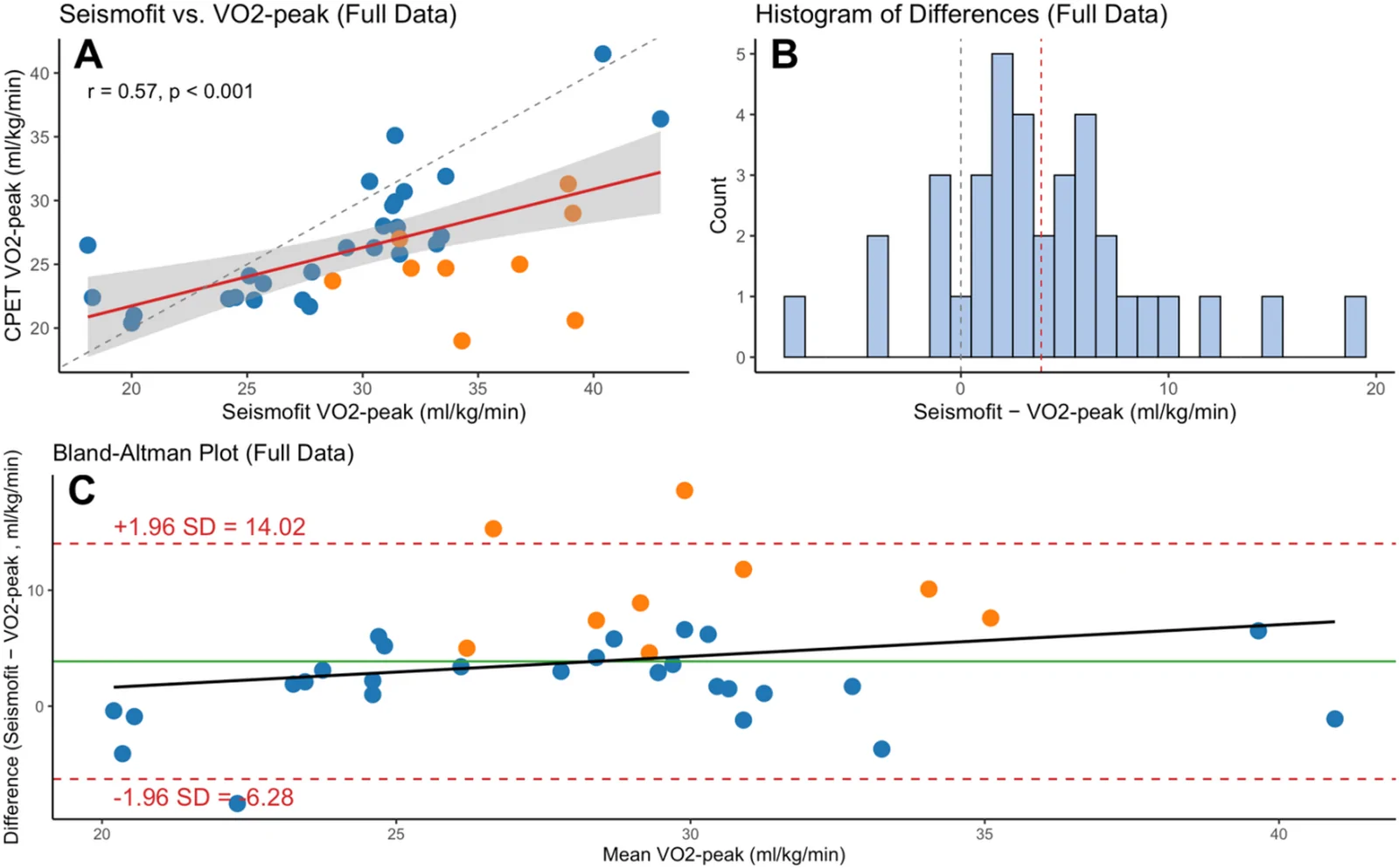
The relationship between the Seismofit-estimated V̇O₂-peak and the gold-standard measure obtained from CPET in the full sample was assessed through their (**A**) correlations; **B** histogram of differences; and **C** Bland-Altman Plot. **A** The scatterplot illustrates the association between Seismofit V̇O₂-peak and CPET-measured V̇O₂-peak, with the red line indicating the observed correlation and the dotted line representing perfect agreement between methods. **B** The histogram shows the spread of differences between Seismofit V̇O₂-peak and CPET V̇O₂-peak. The red line shows the mean difference, and the black line represents a perfect agreement between methods. **C** The Bland-Altman plot shows the mean bias (green line), the correlation (black line), and the 95% limits of agreement (red dotted line). Nine individuals did not reach the V̇O₂ criteria during CPET (orange coloured dots), 9 males and 0 females

**Fig. 2:**
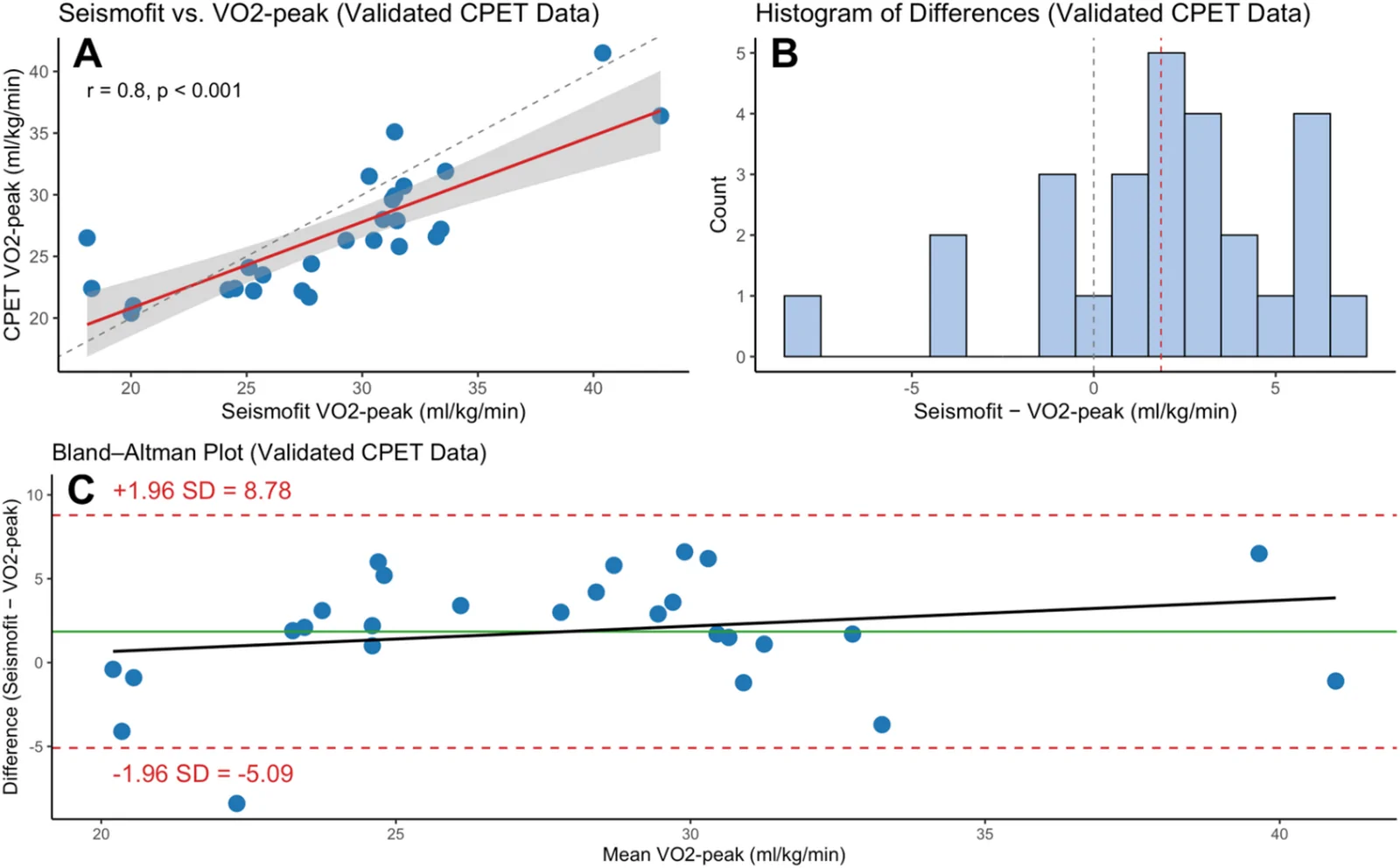
The relationship between the Seismofit-estimated V̇O₂-peak and the gold-standard measure obtained from CPET in the validated CPET sample was assessed through their (**A**) correlations; (**B**) histogram of differences; and (**C**) Bland-Altman Plot. **A** The scatterplot illustrates the association between Seismofit V̇O₂-peak and CPET-measured V̇O₂-peak, with the red line indicating the observed correlation and the dotted line representing perfect agreement between methods. **B** The histogram shows the spread of differences between Seismofit V̇O₂-peak and CPET V̇O₂-peak. The red line shows the mean difference, and the black line represents a perfect agreement between methods. **C** The Bland-Altman plot shows the mean bias (green line), the correlation (black line), and the 95% limits of agreement (red dotted line)

The Pearson correlation for all participants revealed a significant positive correlation between Seismofit and measured V̇O₂-peak (*r* = 0.57, *p* < 0.001; Fig. 1A). When restricting the analysis to participants with a validated V̇O₂-peak, the Pearson correlation showed a significant positive association between Seismofit and measured V̇O₂-peak (*r* = 0.80, *p* < 0.001), see Fig. 2A.

### Bland–Altman analysis

Agreement between CPET and Seismofit V̇O₂-peak was assessed using Bland–Altman analysis. For all participants, the mean bias was 3.87 ml/min/kg, with 95% limits of agreement ranging from − 6.28 to 14.02 ml/min/kg, indicating that Seismofit tends to overestimate V̇O₂-peak relative to CPET (Fig. 1). For participants reaching a validated V̇O₂-peak, the mean bias was 1.85 ml/min/kg, with 95% limits of agreement from − 5.09 to 8.78 ml/min/kg, showing improved agreement and reduced variability (Fig. 2). In the full dataset, three observations were outside these limits, whereas only one was outside when restricting the analysis to validated CPET tests.

### Exploration of sub-groups

The data were stratified into subgroups by sex, age, and fitness level (Supplementary files 1–3).

No significant interaction was observed between Seismofit and sex (*p* = 0.27; Supplementary file 1), age group (*p* = 0.36; Supplementary file 2), or fitness level (*p* = 0.07; Supplementary file 3) in the prediction of V̇O₂-peak .

#### Sex

In stratified analyses, the Pearson correlation in the full dataset showed a significant positive association in males (*r* = 0.49, *p* < 0.05), but not in females (*r* = 0.27, *p* = 0.265). When restricting to the validated CPET sample, both groups demonstrated significant correlations (males: *r* = 0.83, *p* < 0.005; females: *r* = 0.55, *p* < 0.05).

#### Age

For age, both younger (< 70 years) and older (70 ≥ years) participants showed a significant positive correlation in the full (younger; *r* = 0.47, *p* = 0.036; older; *r* = 0.68, *p* = 0.004) and validated CPET samples (younger: *r* = 0.77, *p* = 0.002; older: *r* = 0.84, *p* < 0.001).

#### Fitness Level

For fitness levels, both subgroups (low and high) showed a significant correlation (low V̇O₂-peak; *r* = 0.53, *p* = 0.006; high V̇O₂-peak; *r* = 0.8, *p* = 0.003). When restricting analysis to validated CPET samples, both subgroups (low and high fitness) showed a significant correlation (low V̇O₂-peak; *r* = 0.83, *p* = 0.001; high V̇O₂-peak; *r* = 0.8, *p* = 0.003).

## Discussion

Seismofit has shown promising agreement with V̇O₂-peak in younger adults and athletes, although previous studies have highlighted that further improvement of the model is required for broader clinical application [16, 20]. This study aimed to determine the validity of Seismofit in estimating V̇O₂-peak in older adults. It was hypothesised that SCG-derived V̇O₂-peak estimates would show a strong correlation with gold-standard measured V̇O₂-peak values in healthy older adults.

Seismofit showed a moderate positive association with CPET-measured V̇O₂-peak (*r* = 0.57), which increased to a strong positive correlation when analyses were limited to validated tests (*r* = 0.80), suggesting stronger agreement with CPET V̇O₂-peak in older adults when compared with validated CPET measurements. The Bland–Altman analysis further supported these indications by assessing the agreement between Seismofit and CPET V̇O₂-peak measurements. For both analyses, most differences fell within the limits of agreement. In the full dataset, three observations were outside these limits, whereas only one fell outside when restricting the analysis to validated CPET tests. These limits of agreement are aligned with previous findings in young athletes [20]. Prediction error metrics further indicated moderate agreement between methods, with improved accuracy in the validated subgroup, although individual variability in Seismofit-derived estimates remained. Overall, these results alone do not substantiate the accuracy of the SCG; rather, they suggest a trend towards reasonable consistency with CPET V̇O₂-peak.

The associations observed in our sample (all: *r* = 0.57; validated CPET: *r* = 0.80) were lower, yet comparable, to those reported in previous validation studies of CPET measures in younger adults (*r* = 0.87) and in young athletes (*r* = 0.77) [13,20]. This may reflect age-related differences between young and older adults. Older adults typically exhibit greater variability in cardiac function and lower stroke volume [21], which may influence interpretation of the SCG signal. In addition, obtaining a validated V̇O₂-peak from CPET can be difficult in this population, as many participants are unable to reach true exhaustion due to musculoskeletal limitations or motivational factors [22]. This challenge is well known in ageing cohorts and is one reason large studies often rely on submaximal tests, such as the 6-minute walk test, step tests, or non-exercise predictions based on questionnaires and anthropometric data. The improved agreement between SCG and CPET observed when including only validated CPET tests suggests that the lower correlation in the full sample is likely related to difficulties in achieving a true V̇O₂-peak during CPET, rather than an inaccuracy of the Seismofit measure. However, given that we do not know the true V̇O₂-peak of those with unvalidated CPET data, it is not possible to discard the alternative interpretation that SCG may simply perform better in a selected subgroup of participants. Despite these factors, the strong correlation among participants who reached a validated CPET V̇O₂-peak indicates that Seismofit may offer a valid alternative for estimating V̇O₂-peak in large cohorts of older adults.

In our sample, Seismofit overestimated V̇O₂-peak values. The mean SCG-derived V̇O₂-peak value was 14.6% higher than the CPET-measured V̇O₂-peak, when considering only validated V̇O₂-peak tests, Seismofit reached a higher mean V̇O₂-peak by 4.6% compared to CPET. This systematic bias was further supported by the paired t-test and effect size (Cohen’s d), indicating a consistent overestimation of V̇O₂-peak by Seismofit. Previous studies have found an underestimation of 7% compared to CPET, with a wide limit of agreement in younger adults, indicating a negative bias and high individual variability [16]. This contrast in systematic bias between findings implies that the performance of SCG-based estimation might vary across populations, indicating possible accuracy differences between younger and older adults. A possible explanation for the observed overestimation in our study could be algorithmic bias, which may arise if predictive models are developed on datasets that include mostly younger adults or only a limited number of older adults. Accordingly, age-related changes in cardiovascular performance, pulmonary function, and respiratory mechanics, factors that can influence the physiological signals on which Seismofit’s estimations may rely, may influence the accuracy of Seismofit in older populations [23]. Despite these potential explanations for SCG’s tendency to overestimate V̇O₂-peak relative to CPET, the systematic overestimation may represent a significant limitation of Seismofit within the older population and should be taken into account when interpreting SCG-estimated V̇O₂-peak in both clinical and research contexts.

### Exploratory sub-group analyses

To explore tendencies in how well Seismofit estimates may vary by sex, age group, and fitness level, factors known to influence V̇O₂-peak measured with CPET, as well as interactions and sub-group differences, were examined. Of note, none of the interactions were significant, and results from the subgroup analyses are presented to generate hypotheses for future examinations. The association between Seismofit and CPET-measured V̇O₂-peak was not found to vary by sex, age group, or fitness level. In studies examining sex differences in cardiorespiratory fitness using Seismofit, no significant difference in the accuracy has been observed between young men and women [24]. We observed that, when examining sex differences, Seismofit in males showed a significant association with CPET-measured V̇O₂-peak, while no significant association was observed in females. However, when the analysis was restricted to participants with validated CPET measurements, Seismofit showed an association in both sexes. Importantly, while the sub-group differences between males and females could be of interest for future investigation, these findings should be interpreted with caution given the limited sample and exploratory nature of this analysis.

### Limitations

This study has some limitations that should be acknowledged. Importantly, the small sample size reduces reliability, generalizability, and power for subgroup analyses and may cause variability in agreement estimates. Furthermore, this study relies on correlation coefficients to characterize the relationship between Seismofit- and CPET-derived V̇O₂-peak. While the correlation values ranged from moderate to strong, correlation coefficients alone do not establish agreement or interchangeability between the methods. Consequently, a high correlation should not be taken as evidence of sufficient accuracy, particularly considering the observed limit of agreement that reveals the residual variability between the two methods. Another limitation is that each participant only had one Seismofit and one CPET measurement; repeating Seismofit assessments across sessions could improve reliability and account for day-to-day variability. Of note, a recent study using seismocardiography has shown that repeated measurements can capture individual-level V̇O₂-peak changes [20]. A limitation of the present study is that it only evaluates baseline V̇O₂-peak agreement between CPET and Seismofit and does not assess longitudinal responsiveness, which has previously been shown to be limited for Seismofit methods in trained populations [25]. It should also be noted that the study sample was recruited from the Danish population in the Greater Copenhagen area, which could be argued to not be representative of the wider population of Danish older adults. However, in contrast to the expected healthy bias, the fitness level of our sample was lower than the age-matched fitness levels reported in a large sample of Danish older adults [15]. The tendency to recruit healthy older adults in scientific studies may not be a limitation in terms of validating Seismofit within our sample, but it does make it more difficult to generalize the validation of the device to the broader population of older adults.

## Conclusion

Overall, the findings of this study demonstrate that Seismofit provides an estimate of V̇O₂-peak that shows a moderate-to-strong association with CPET measurements in older adults. However, Seismofit overestimated V̇O₂-peak in older adults, and its systematic bias should be considered. Further development and refining of this methodology in ageing populations could facilitate the inclusion of fitness level data in large-scale cohort studies and experimental research.

## Supplementary Information


Supplementary Material 1.


## Data Availability

The data that support the findings of this study are not openly available due to reasons of sensitivity and existing data protection agreements. Secondary data are available from the senior author upon reasonable request. Data are located in controlled access data storage at the Copenhagen University Hospital - Amager and Hvidovre.

## Abbreviations

CPET: Cardiopulmonary exercise test
V̇O₂-peak: Peak oxygen uptake
RER: Respiratory exchange ratio
CRF: Cardiorespiratory fitness
SCG: Seismocardiography
MRI: Magnetic resonance imaging
ECG: Electrocardiogram
HINT: HIT-ing the neurometabolic targets for exercise-induced effects on cognition
PASE: Physical activity scale for the elderly
RPM: revolutions per minute
MAPE: mean absolute percentage error
SEE: The standard error of the estimate
BPM: Beats per minute
