# Supplementary material for: Seismocardiography for nonexercise estimation of cardiorespiratory fitness in older adults
Source: BMC Geriatr. 2026 Jun 20;26:1121. doi: 10.1186/s12877-026-07771-6 (PMC13536557; doi:10.1186/s12877-026-07771-6)
Supplement: Supplementary file 1 [file 12877_2026_7771_MOESM1_ESM.docx]

**Supplementary Files**

*Supplementary file 1: Participant Characteristics and Fitness Measurements for the sex subgroups*

| Participant Characteristics | Female (n =19) | Male (n=17) | |
| --- | --- | --- | --- |
| Age (years) | 70.5 ± 3.0 | 68.9 ± 3.0 | |
| Height (cm) | 161.9 ± 5.4 | 175.5 ± 5.7 | |
| Weight (kg) | 64.9 ± 11.1 | 82.3 ± 13.4 | |
| BMI (kg/m2) | 24.8 ± 4.4 | 26.6 ± 3.0 | |
| Fitness Measurements |  |  | |
| Maximum Heart Rate (BPM) | 151.4 ± 9.6 | 158.8 ± 10.3 | |
| V̇O₂-peak (ml/min/kg) | 24.3 ± 3.1 | 28.9 ± 5.4 | |
| RER | 1.3 ± 0.1 | 1.3 ± 0.1 | |
| Seismofit (ml/min/kg) | 27.0 ± 4.6 | 34.0 ± 5.3 | |
| PASE | 111.0 ± 42.2 | 158.8 ± 58.2 | |

*Supplementary file 2: Participant Characteristics and Fitness Measurements for the age subgroups*

| Participant Characteristics | Under 70 years (n =20) | 70+ years (n=16) | |
| --- | --- | --- | --- |
| Age (years) | 67.3 ± 1.3 | 72.8 ± 1.3 | |
| Height (cm) | 169.2 ± 7.7 | 167.2 ± 10.1 | |
| Weight (kg) | 72.1 ± 13.0 | 74.4 ± 17.5 | |
| BMI (kg/m2) | 25.1 ± 4.1 | 26.3 ± 3.5 | |
| Fitness Measurements |  |  | |
| Maximum Heart Rate (BPM) | 156.9 ± 11.7 | 152.3 ± 8.4 | |
| V̇O₂-peak (ml/min/kg) | 27.4 ± 5.2 | 25.3 ± 4.3 | |
| RER | 1.2 ± 0.1 | 1.3 ± 0.1 | |
| Seismofit (ml/min/kg) | 32.5 ± 6.3 | 27.6 ± 4.5 | |
| PASE | 136.4 ± 53 | 125 ± 56.1 | |

*Supplementary file 3: Participant Characteristics and Fitness Measurements for the fitness level subgroups*

| Participant Characteristics | Low fitness level (n=25) | High Fitness level (n= 11) | |
| --- | --- | --- | --- |
| Age (years) | 69.5 ± 2.9 | 70.4 ± 3.3 | |
| Height (cm) | 168.1 ± 9.2 | 168.9 ± 8.0 | |
| Weight (kg) | 72.9 ± 15.2 | 73.5 ± 14.8 | |
| BMI (kg/m2) | 25.6 ± 3.6 | 25.7 ± 4.5 | |
| Fitness Measurements |  |  | |
| Maximum Heart Rate (BPM) | 153.7 ± 10.1 | 157.6 ± 11.2 | |
| V̇O₂-peak (ml/min/kg) | 24.6 ± 3.3 | 30.7 ± 5.3 | |
| RER | 1.3 ± 0.1 | 1.28 ± 0.08 | |
| Seismofit (ml/min/kg) | 30 ± 5.9 | 31.1 ± 6.7 | |
| PASE | 132.7 ± 62.1 | 127.2 ± 41.7 | |
